# Effects of Nanocrystal Cellulose from Bamboo on the Flexural Strength of Acrylic Resin: In Vitro

**DOI:** 10.3390/dj10070129

**Published:** 2022-07-07

**Authors:** Visakha Aupaphong, Kriangsak Kraiwattanawong, Bhornsawan Thanathornwong

**Affiliations:** 1 Faculty of Dentistry, Thammasat University, Pathum Thani 12120, Thailand; aupaphon@tu.ac.th; 2 Department of Chemical Engineering, School of Engineering, King Mongkut’s Institute of Technology Ladkrabang, Bangkok 10520, Thailand; kriangsak.kr@kmitl.ac.th; 3 Faculty of Dentistry, Srinakharinwirot University, Bangkok 10110, Thailand

**Keywords:** dental materials, acrylic resins, denture bases, flexural strength, nanoparticles

## Abstract

The purpose of this study is to evaluate the effects of nanocrystal cellulose (NCC) from bamboo on the flexural strength of heat-cured acrylic resin. A total of 35 specimens (3.3 mm × 10 mm × 64 mm) were prepared and the specimens were divided into five groups of seven specimens each. Group 1 used conventional acrylic resin that was prepared based on the instructions of the manufacturer (0%). The filled NCC from bamboo fiber in four concentrations (0.25, 0.5, 1, and 2% *w*/*w*) was used in the four-reinforcing resin workpiece groups. The specimens were loaded until failure occurred on a three-point bending test machine. One-way analysis of variance and Dunnett’s multiple comparison test at a 95% confidence level were used to determine the statistical differences in the flexural strength among the five groups. The results found that the average flexural strength of five specimen groups (0, 0.25, 0.5, 1, and 2% *w*/*w*) were 60.11 ± 2.4, 60.75 ± 2.18, 66.50 ± 5.08, 56.04 ± 0.31, and 48.05 ± 2.61 MPa, respectively. The flexural strength of 0.5 mg% *w*/*w* NCC-reinforced acrylic resin was significantly higher than the control group (*p* < 0.01). The reinforced NCC from bamboo fiber to acrylic resin improved the flexural strength properties.

## 1. Introduction

The denture base is the part of a denture that rests on the foundation tissues and to which prosthetic teeth are attached. A complete or partial denture base can be used to replace missing natural teeth. The poly-methyl methacrylate (PMMA) is the most commonly used material for the fabrication of denture bases and has a number of advantages [1,2]. The methyl methacrylate resin denture base is distinguished by its clarity, relatively high level of strength and hardness, color stability under all chemical conditions of oral functions, and insolubility in mouth fluids. All of these characteristics were combined to create a material suitable for prosthetic dentures [1,2,3].

Despite the excellent physical properties of PMMA, the most common complaints to dentists from denture wearers is a fractured denture [4]. The acrylic resin base fractures occurred most often due to occlusal force or accidental damage. The flexural strength of resin materials is one of the most critical mechanical properties leading to fracturing dentures [5]. Flexible partial dentures have recently gained popularity as an option for people who are not able to wear traditional dentures. However, they may not be capable of supporting a healthy bite. These dentures may just not be strong enough to support a bite in a good position. Therefore, perfection in the mechanical performance of denture base structures was attempted by adding a reinforcing component to the PMMA matrix, raising the force resistance of denture base resins [6].

People have become increasingly concerned about environmental issues in the twenty-first century. NCC is a type of sustainable natural resource. It is a rod-shaped monocrystalline cellulose, with a length of tens to hundreds of nanometers and a diameter of one to hundreds of nanometers. Due to its availability, lightweight and unique shape, nanoscale dimensionality, and unsurpassed intrinsic physical and chemical qualities, it has been the subject of a wide range of research activities. NCC has received a lot of interest for its possible usage following the reinforcement and retention of polymer composites because of its high Young’s modulus and unique tensile strength [7,8]. It is noteworthy that the elastic modulus of bamboo fiber with NCC reinforcements was determined to be 94.1 and 127.3 for NCC concentrations of 1 and 3 wt%, respectively [9]. 

The main natural source of cellulose is plant fibers. They are complex biocomposite structures that occur naturally. A single cell is a fundamental plant fiber. A single fiber resembles a microscopic hollow tube with a core lumen surrounded by the cell wall. Plant cell walls can be used to extract the NCC, which is a nanometer-sized substance. NCC has a high strength value in its properties. It is tough, with a greater surface area, and both inexpensive and biodegradable. There were also surfaces with a significant number of hydroxyl groups that were appropriate for adherence to polarized substance groups. NCC is currently employed in a variety of goods, including medical devices, textiles, restorative materials, and many others [10]. 

The use of renewable natural fibers as reinforcements in composite materials has grown in significantly in recent years. Reinforced plastics containing cellulosic materials as fillers are low-cost, lightweight, and hazard-free, and have improved mechanical qualities. The various fiber species have different mechanical qualities due to their function in nature, reflected in their physical, chemical, and morphological characteristics. The cellulose content, for example, is primarily responsible for tensile strength, and the microfibrillar angle was proportional to the strain-to-failure due to irreversible cell wall deformation. The cellulose concentration is proportional to the modulus of elasticity, which is inversely proportional to the microfibrillar angle. There have been studies on plastics reinforced with natural fibers such as siwak, bamboo, sisal, pineapple leaf, and wood fibers [4,11,12,13,14]. The handling process for incorporating NCC (hydrophilic) in non-polar polymers is difficult since the components usually have poor adhesion, resulting in weak interfacial bonding. For related nanocomposite synthesis, there are three options: (a) dispersing dry cellulose into a hydrophobic matrix, (b) dispersing cellulose and the polymer matrix in the common solvent, or (c) employing aqueous nanocellulose dispersion [15].

Bamboo is a plant that is both easy to discover and cultivate, and is a good source of natural cellulose. The mechanical characteristics of bamboo fiber are reviewed by Liu D. et al. [11]. Bamboo is well known for its superior mechanical qualities, due to its principal element, fiber, and the unidirectional arrangement of the fiber in the tissue. Thwe et al. [4] studied the effect of bamboo fibers on tensile properties of self-cured acrylic resin used for denture applications. The result found that the tensile strength and Young’s modulus increased alongside an increase in the weight fraction and the fiber length of the reinforcing bamboo in PMMA resin. The bamboo cellulose fibrils in the fiber walls were virtually axially aligned, maximizing the longitudinal elastic modulus of the fibers, while lignification increased their transverse rigidity.

The influence of nanocrystalline cellulose on the tensile qualities and the properties of self-cured acrylic resin used for denture application were studied. The fiber length and concentration of bamboo fiber enhanced the tensile strength and Young’s modulus, and also improved the hardness, compression, and concentration rate of bamboo powders in composite samples [16,17].

Bamboo has a good toughness, hygroscopicity, crystallinity, and a great potential for generating NCC. The geometrical dimensions of NCC can vary widely, with a diameter in the range of 5–50 nm and a length in the range of 100–500 nm. The dimensions and crystallinity of a given NCC depend on the cellulose source and extraction conditions [18,19]. NCC from bamboo fiber may play an important role in forming future organic structures and composites, and is recognized as an attractive candidate for strengthening natural fibers [20,21,22]. However, previous studies have mentioned bamboo microfibril, but the character and property of NCC from bamboo cooperated with an acrylic denture base have not been investigated, especially with regards to their flexural strength. Therefore, the purposes of this study were to evaluate the effects of NCC from bamboo on the flexural strength of acrylic resin. The hypothesis of this study was that adding NCC from bamboo to acrylic resin increased the flexural strength.

## 2. Materials and Methods

### 2.1. Bamboo NCC Preparations

- Pre-treatment of bamboo fibers

The bamboo fiber chips were provided by a commercial retailer. They were dried for 24 h in a hot air oven at 60 °C. Then, 10 g of dried bamboo fiber chips was stirred in 1000 mL of distilled water at 80 °C for one hour, to remove any foreign materials, such as pectin. The solution of bamboo fiber was sieved out. The fibers were then incubated for 24 h at 70 °C to eliminate the fatty acids, and 1 g of dry bamboo fiber was processed in 30 mL of 20% NaOH solution at 80 °C for 2 h. The solution was then filtered using a vacuum filter. The bamboo fiber residuals were washed with distilled water. Next, the treated bamboo fibers were incubated for 24 h at 70 °C. Then, they were bleached in a 1:1 ratio of 25% H_2_O_2_ and 5% NaOH for one hour. The solution was heated to 70 °C. This process was performed six times until the bamboo fiber was white, and after that they were kept in a 70 °C incubator for 24 h [22].

- NCC extraction

**Figure 1:**
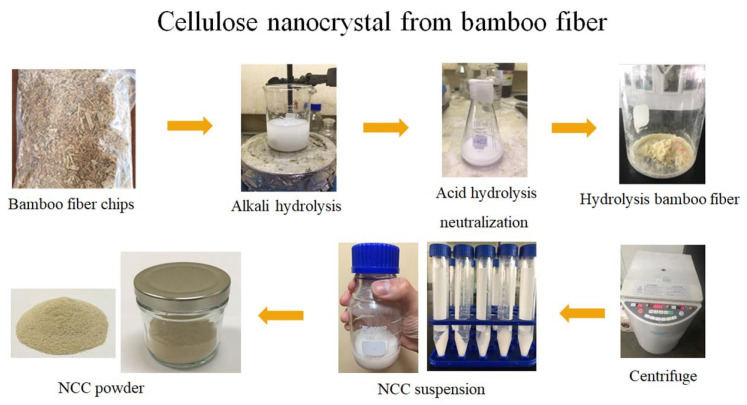
Process of nanoparticle crystal preparation from bamboo.

**Figure 2:**
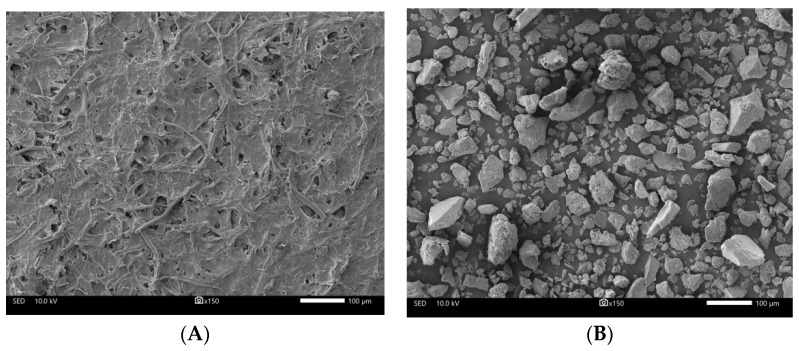
(**A**) SEM image the NCC suspension in water. (**B**) NCC powder prior to sieving.

The treated bamboo fibers were hydrolyzed using 300 mL of 64% sulfuric acid and stirred continuously at 45 °C for two hours. When the suspensions became dark yellow, they were neutralized with distilled water to stop the hydrolysis reaction and then centrifuged for 10 min at 10,000 rpm. After allowing the solutions to settle for several hours, the suspensions were layered and the tops were clear. The clear tops were removed and the remaining solution was repeatedly rinsed with distilled water, until the tops were no longer layered [22]. The NCC suspension was freeze-dried to produce NCC powder, which was then incubated at 110 °C to eliminate any moisture. A standard mesh sieve was then used to sieve NCC coarse powder (Figure 1). The NCC suspension in water and NCC powder were characterized using a scanning electron microscope (SEM). SEM images of NCC revealed that the rod-like structure of the crystallite had a length ranging from 200 nm to several micrometers (Figure 2).

### 2.2. Workpiece Preparation

**Figure 3:**
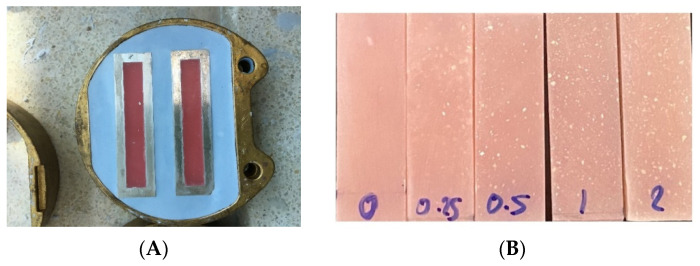
(**A**) The stainless-steel workpiece mold. (**B**) The five groups of NCC-reinforced acrylic resin workpieces.

The dental wax (Tenatex Red, Kemdent, UK) was placed in 3.3 × 10 × 64 mm^3^ stainless-steel patterns and then all of the components were placed in a metal flask with a dental stone (Bego, Bremen, Germany) and processed to remove the wax pattern, resulting in the creation of a stainless-steel mold for acrylic resin workpieces (Figure 3A). Before placing acrylic resin in the mold, the stainless-steel mold surfaces were treated with separating media (Apollon Sep, Aichi, Japan).

The sample size was calculated using the free G*power3.1.9.7 software for five groups, resulting in a total of 35 samples. Group 1 served as a control group, using a standard acrylic resin (Meliodent, Hessen, Germany) and based on the instructions of the manufacturer (0%). The chemical composition of this acrylic resin comprised polymer powder (polymethylmethacrylate, ethyl hexyl acrylate, and N-octyl methacrylate) and monomer liquid (methyl methacrylate, glycol dimethacrylate, and dimethyl p-touludine). The pilot study had an experimental concentration of NCC (data not shown), and the NCC was used to add the acrylic resin for Groups 2–5 in four different concentrations: 0.25, 0.5, 1, and 2% *w*/*w*, respectively. NCC powder was weighed and mixed with acrylic powder as previously described, and then the monomer was added in mixtures and became homogeneous by manual stirring. 

All of the workpieces were put into the stainless-steel mold flasks during the dough stage and hydraulic-pressed using the conventional method. To complete the polymerization cycles, the flasks were cured in a water bath at 72 °C for one hour, and then at 100 °C for 30 min to achieve polymerization according to the recommendations. Before the workpieces were removed, the flasks were placed in the water overnight. After cooling to room temperature, the specimens were deflasked, and the excess material was removed using a carbide bur in a low-speed rotary instrument. The Nano 1000 S polisher was used to finish the workpieces (Figure 3B).

### 2.3. Three-Point Flexural Strength Test

**Figure 4:**
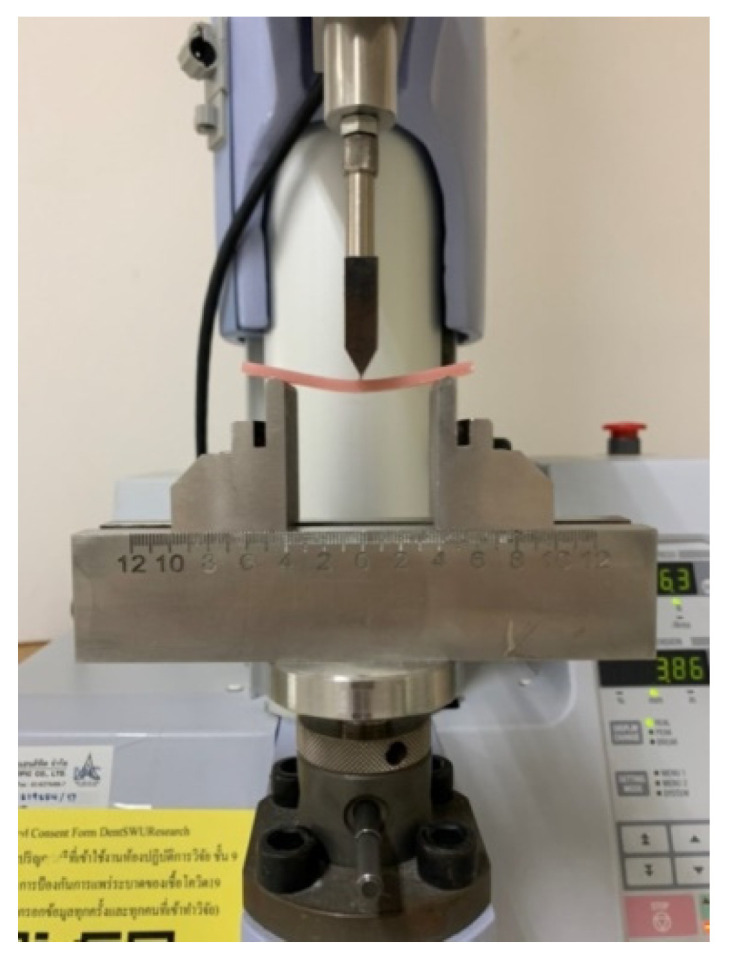
The testing machine and resin workpieces during the three-point flexure test.

The international standards on polymer materials, such as ISO 1567:1999, have adopted a three-point flexural test. The Dentistry Denture base polymer is the most popular method of determining the flexural properties of denture bases [23]. The 35 workpieces were subjected to a three-point flexural strength test, with seven samples in each group. The force was applied by the universal testing machine (Shimadzu Ez-L, Kyoto, Japan) (Figure 4) with TRAPEZIUM X software and a 500 N load cell at a crosshead speed of 2 mm/min. The span length was 46 mm. 

The flexural strength (FS, in MPa) was calculated by FS = 3PL/2 bd2, where P is the maximum load (N), L is the span length (m), b is the specimen width (m), and d is the specimen thickness (m) [24]. After the flexural testing, the NCC from the bamboo fiber in workpieces was characterized using a SEM.

### 2.4. Statistical Analysis

The data collection showed an average ± standard deviation (SD) of flexural strength and the analysis of the study was performed using SPSS software, Version 21.0. All of the data were determined for normal distribution using the Kolmogorov–Smirnov normality test. The differences between flexural strength were analyzed using one-way analysis of variance and Dunnett’s multiple comparison test at the 95% confidence level.

## 3. Results

### 3.1. Fractural Strength of NCC-Reinforced Acrylic Resin

**Figure 5:**
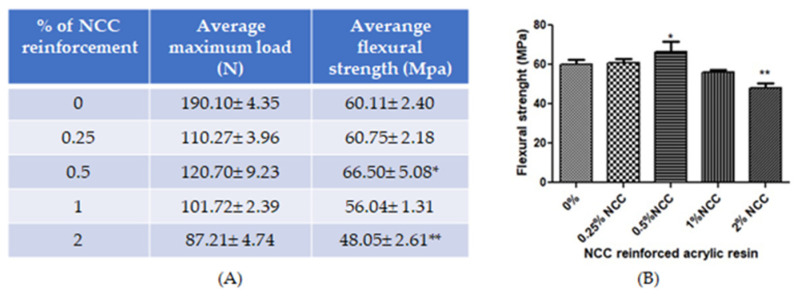
(**A**) The average and SD of maximum load and fractural strength of NCC-reinforced acrylic resin (**B**) The graph of the average and SD fractural strength of NCC-reinforced acrylic resin * *p* < 0.05, ** *p* < 0.005 vs. 0% (N = 7).

The average flexural strength with SD is shown in Figure 5. The results revealed that the 0.5 mg% *w*/*w* NCC-reinforced acrylic resin had the highest flexural strength at a mean flexural strength around 66.50 ± 5.08 MPa, followed by 0.25 mg%, 0%, 1%, and 2% *w*/*w*, respectively. The 0.5 mg% *w*/*w* NCC-reinforced acrylic resin had significantly higher flexural strength than the control (0%), while the 2 mg% *w*/*w* NCC-reinforced acrylic resin had significantly lower flexural strength.

### 3.2. SEM of NCC-Reinforced Acrylic Resin

**Figure 6:**
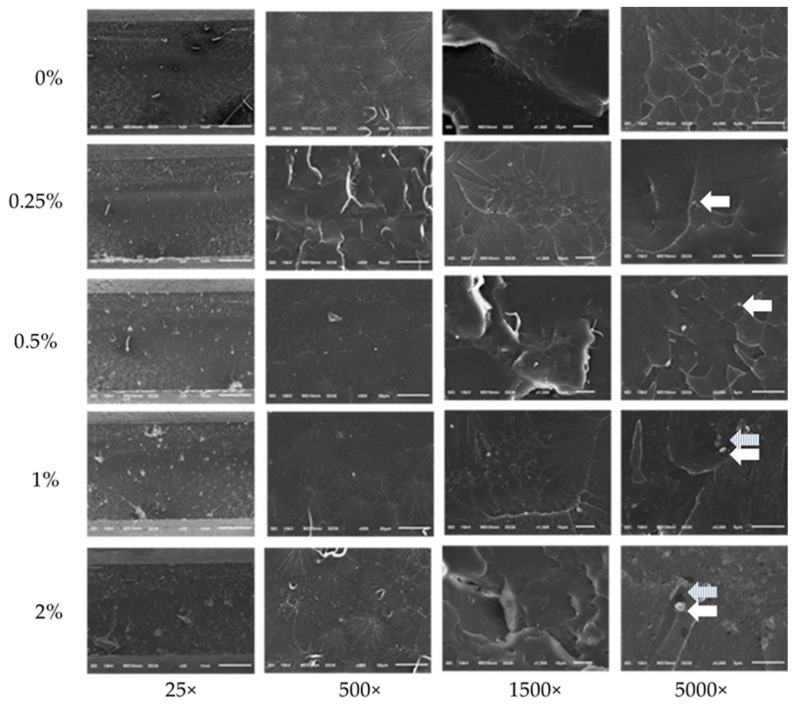
Fractured workpieces under SEM (white arrow: NCC, stripe arrow: the space between the fiber and the matrix).

After flexural strength testing, all of the fractures in the 35 workpieces were characterized by the microscopic features of SEM using a 25×, 500×, 1500×, and 5000×, respectively. It was evident that the nanocellulose from bamboo fiber was distributed in a collective and scattered insertion in a matrix resin (Figure 6). 

## 4. Discussion

This study focused on the NCC from bamboo fiber on the flexural strength of the heat-cured acrylic resin. According to the findings, the present study confirmed that NCC from bamboo increased the flexural strength in concentrations of 0.25 and 0.5% *w*/*w*. The results showed a rise in flexural strength in a dose-dependent pattern for proportions of 0, 0.25, and 0.5% *w*/*w*. From SEM, 0.25 and 0.5% *w*/*w* specimens were homogeneous with a denture matrix and less void when compared to conventional acrylic resin. The use of the mixture of polymer powder and the monomer liquid of acrylic resin was important to avoid an excess of monomers. When acrylic resin polymer powder and monomer liquid were mixed, monomers dissolved and swelled the surface of the powder beads. Subsequently, after mixing the powder and liquid, small quantities of “free” monomers were left to penetrate into the porous polymer, resulting in weak acrylic resin. The CNN from bamboo filler used in the studies may be directly responsible for a copolymer and may be taken into polymer reinforcements. As mentioned by Reis et al. [23], the strength related to the improved interfacial strength between nanoparticles and a matrix created by crosslink bonding covered the NCC. 

However, adding more NCC to bamboo fiber by 1% and 2% percent *w*/*w* reduced the average flexural strength, even if the SEM picture of the workpieces did not alter noticeably and because of the 2% *w*/*w* NCC from bamboo fiber. It was smaller than the control groups at a statistically significant level, making it likely that the excess space between the fiber and the matrix was caused by the filler deposited between the polymer and the fiber. As a result, the interfacial strength was poor. If force was applied to the workpieces, the area would fracture [4,24,25].

In comparison to Oleiwi JK et al. [16,17], the results showed that increasing the bamboo fiber powder weight by 3, 6, and 9% increased the compressive and tensile strength of polymethyl methacrylate. The tensile strength of reinforcing fibers increased somewhat as the weight percent of reinforcing fibers increased. This is due to the strengthening mechanism of the reinforcing fibers, in which the quantity of these fibers plays a significant role in impeding. The slipping of PMMA resin chains also increased. It is worth mentioning that bending between the fibers and chains in a little space required a lot of stress. The interface bonding between the reinforcing fibers and the matrix was also important. Moreover, the interface bonding between the reinforcing fibers and matrix was an essential part, so the results of the composite demanded a high value of stress to break their interface bonding. The compression strength values for natural bamboo increased as the weight fraction increased for 25 µm and 75 µm particle sizes. The ability of powder to strengthen the matrix, improve mechanical bonding between powder and matrix (PMMA), and improve mechanical properties associated with the addition of natural particles with high compression strength compared to PMMA matrix all contributed to an increase in the compression strength. From the pilot study, the concentration of NCC was more than 2% *w*/*w* and clearly decreased the flexural strength as a result of weak interfacial bonding. A higher percentage resulted in a greater effect and lowered the mechanical performance of the composite [26,27].

The compressive strength is the force responsible for the deformation of the material, such as the volume of the reduced material, whereas the tensile strength refers to the ability of a substance to withstand tension [2]. It is not the same phenomenon as chewing force, which occurs repeatedly and in multiple directions. When food is trapped between artificial teeth, the force acts on both sides of the denture, causing the central area to bend, similar to flexural strength testing [28]. Many of these fractures develop inside the mouth as a result of mastication-induced denture fatigue [29]. It is well acknowledged that many materials lose strength over time as a result of cyclical stress. These are caused by lateral fiber spreading when acrylic resin dough is pressed into the mold, which reduces the concentration of fibers in a polymer matrix, resulting in non-homogeneous fiber distribution in the matrix and poor fiber wetting by acrylic resin material. The layer of acrylic resin surrounding the single fibers in the middle of the fiber roving was not even, and polymerization shrinkage such as PMMA has been identified as a cause of complete denture midline fractures. It destroyed the layer of acrylic resin on the surface fibers and decreased the bond between the fibers and the polymer material [30]. 

Denture fractures are the most common problem and, alternately, microcracks may begin to form. Stresses in the denture propagate through the material, eventually resulting in fatigue failure after a period of time. Therefore, the fatigue test of NCC from bamboo-reinforced resin should be evaluated next. Furthermore, acrylic resin dentures flexed significantly more than expected during function, and poor tissue adaptation was observed [31].

Particles, wires, fibers, or mesh aligned with the shape of the denture base have all been attempted to improve the mechanical qualities of denture-based acrylic resins. However, previous studies did not utilize bamboo fiber nanoparticles to cooperate with acrylic resin; thus, this study attempted to test the property of the nanoparticle effect on acrylic resin. The results corresponded to Salman and the college study [25]. They compared crystalline and amorphous nanosilica impact and flexural strength, which was incorporated into a polymethyl methacrylate denture base. The lowest concentration of amorphous nanosilica (7% by weight) had the highest flexural strength and decreased a higher concentration. Though the crystalline nanosilica at 5% had the highest flexural strength, it also decreased following a higher concentration. However, increasing flexural strength that did not relate to compressive strength also increased. The highest impact strength was 3% of amorphous nanosilica and crystalline nanosilica. Thus, the optimum percentage has to balance both flexural and impact strength [4,16,17,25]. Furthermore, alterations in tissue support for dental dentures resulted in the base of the dental dentures not adhering to the surface of the tissue. It can cause the base of the denture to flex and eventually crack. According to this study, the solution had a higher flexural strength value than the basis of traditional dental dentures. In order to meet the standard of the dimensional stability measures of the NCC, reinforced dentures should be tested while immersed in water.

This study revealed a lower flexural strength compared to previous reports. Golbidi et al. [32] evaluated of the flexural properties of Meliodent heat-polymerized acrylic resins. The results found that the flexural strength was 81.548 (±1.541), which was higher than the control group in this study. However, several factors can influence flexural strength, such as the degree of polymerization; powder particle size; porosity; polymer molecular weight; and the amount of residual monomer, fillers, and plasticizers [2,12]. It has been suggested that shorter polymerization cycles have been reported to increase the amount of residual monomer [33]. The plasticizing effects of excess monomer may decrease the flexural strength of acrylic resins.

The reinforced NCC made from bamboo fibers at the proper rate increased the flexural strength of the acrylic resin denture base. There was also crucial knowledge about how to improve the mechanical properties of acrylic resin; thus, the findings of the study can be applied to future research on how to improve the flexural strength of prosthetic bases. NCC made from bamboo fibers as a reinforced material can be used as an alternative to increase the flexural strength of acrylic denture foundation.

This study was limited to a mechanical test to determine the flexural strength of NCC from bamboo on acrylic resin, and it is still unclear if NCC is suitable for use as a filler. As a result, it appears that testing a longer polymerization cycle on reinforced NCC made from bamboo fiber acrylic resins requires more research. Further investigation of the effects of parameters, such as other mechanical tests, polymer molecular weight, porosity, and aesthetics, are recommended in order to make further assessments.

## 5. Conclusions

The addition of 0.5 percent of reinforced NCC from bamboo fiber to acrylic resin significantly increased flexural strength properties. 

## Data Availability

Not applicable.
